# Hermes Regulates Axon Sorting in the Optic Tract by Post-Trancriptional Regulation of Neuropilin 1

**DOI:** 10.1523/JNEUROSCI.2400-16.2016

**Published:** 2016-12-14

**Authors:** Hanna Hörnberg, Jean-Michel Cioni, William A. Harris, Christine E. Holt

**Affiliations:** Department of Physiology, Development and Neuroscience, University of Cambridge, Downing Street, Cambridge CB2 3DY, United Kingdom

**Keywords:** axon sorting, RNA-binding protein, topography, visual system

## Abstract

The establishment of precise topographic maps during neural development is facilitated by the presorting of axons in the pathway before they reach their targets. In the vertebrate visual system, such topography is seen clearly in the optic tract (OT) and in the optic radiations. However, the molecular mechanisms involved in pretarget axon sorting are poorly understood. Here, we show in zebrafish that the RNA-binding protein Hermes, which is expressed exclusively in retinal ganglion cells (RGCs), is involved in this process. Using a RiboTag approach, we show that Hermes acts as a negative translational regulator of specific mRNAs in RGCs. One of these targets is the guidance cue receptor Neuropilin 1 (Nrp1), which is sensitive to the repellent cue Semaphorin 3A (Sema3A). Hermes knock-down leads to topographic missorting in the OT through the upregulation of Nrp1. Restoring Nrp1 to appropriate levels in Hermes-depleted embryos rescues this effect and corrects the axon-sorting defect in the OT. Our data indicate that axon sorting relies on Hermes-regulated translation of Nrp1.

**SIGNIFICANCE STATEMENT** An important mechanism governing the formation of the mature neural map is pretarget axon sorting within the sensory tract; however, the molecular mechanisms involved in this process remain largely unknown. The work presented here reveals a novel function for the RNA-binding protein Hermes in regulating the topographic sorting of retinal ganglion cell (RGC) axons in the optic tract and tectum. We find that Hermes negatively controls the translation of the guidance cue receptor Neuropilin-1 in RGCs, with Hermes knock-down resulting in aberrant growth cone cue sensitivity and axonal topographic misprojections. We characterize a novel RNA-based mechanism by which axons restrict their translatome developmentally to achieve proper targeting.

## Introduction

In the vertebrate visual system, retinal ganglion cells (RGCs) project topographically to the optic tectum (superior colliculus in mammals) along the anterior–posterior (A-P) and dorsal–ventral (D-V) axis. This topography is established, as first postulated by Roger Sperry (Sperry, 1963), by the graded expression of guidance cues (e.g., Ephrins) at the target, which guide RGC axons that express graded amounts of the receptors (Ephs) for these cues (Feldheim and O'Leary, 2010). Interestingly, however, RGC axons are already topographically sorted in the optic tract (OT) before reaching their target (Scholes, 1979; Fawcett et al., 1984; Stuermer, 1988; Plas et al., 2005). This sorting occurs only after the axons have crossed the optic chiasm, indicating that it is regulated precisely. Differential expression of Neuropilin 1 (Nrp1) and Semaphorin3A (Sema3A) have been shown to regulate the correct pretarget topographic position of axons in the olfactory system and corpus callosum (Imai et al., 2009; Zhou et al., 2013).

Post-transcriptional regulation has emerged as a key mechanism in the temporal regulation of protein expression in axons during development (Holt and Schuman, 2013; Batista and Hengst, 2016; Jain and Welshhans, 2016). RNA-binding proteins (RBPs) play a crucial part in this process (Hörnberg and Holt, 2013) and several RBPs have been implicated in axon guidance and target recognition (McWhorter et al., 2003; Yao et al., 2006; Li et al., 2009; Glinka et al., 2010; Ymlahi-Ouazzani et al., 2010; Welshhans and Bassell, 2011). Of particular relevance to this study is work by Chien and colleagues showing that the fragile X-interacting protein CYFIP2 (cytoplasmic FMR1 interacting protein 2), a protein linked to RNA regulation, causes tract-sorting errors of dorsal axons in the OT (Pittman et al., 2010). This result indicates that translational regulation may be involved in the sorting of RGC axons. We have shown previously that the RBP Hermes (RBPMS, RNA-binding protein with multiple splicing) is expressed exclusively in RGCs during and after axogenesis, but its mechanism of action is unknown (Hörnberg et al., 2013).

Here, we investigated the role of Hermes during axon sorting in the OT of zebrafish and *Xenopus laevis*. We found that Hermes knock-down induced topographic errors in the positioning of dorsal axons in the OT, which led many to enter the tectum medially rather than laterally. We identified several mRNAs with expression that was specifically downregulated by Hermes in RGCs, including the Semaphorin3A-sensitive guidance receptor Neuropilin1 (Nrp1). Reducing excessive Nrp1 activity in Hermes-depleted zebrafish embryos was sufficient to restore correct topographic sorting of dorsal axons in the OT. Together, these findings demonstrate a critical role for Hermes as a translational regulator during topographic map formation in the visual system.

## Materials and Methods

### Embryo maintenance

*Xenopus* embryos were obtained by *in vitro* fertilization, raised in 0.1 × modified Barth's saline (MBS) at 14–20°C, and staged according to the tables of Nieuwkoop and Faber (1967). Zebrafish embryos of either sex were obtained from WT (AB-TL or TL) strains that were maintained and bred at 26.5°C. The stable transgenic atoh7:rpl10a-GFP line was obtained using the Tol2 system (Suster et al., 2009). Embryos were raised at 28.5°C in E3 embryo medium. Embryos to be collected for fluorescence imaging had the embryo medium supplemented with 0.003% phenylthiourea (Sigma-Aldrich) for pigment reduction. All animal work was approved by local ethical review committee at the University of Cambridge and was performed according to the protocols of project license PPL 80/2198.

### DNA constructs and morpholinos (MOs)

All constructs were expressed in the pCS2+ vector (David Turner, University of Michigan, Ann Arbor). *Xenopus* hermes cDNA (GenBank GeneID AF107889) was kindly provided by Dr. M. Kloc and sublconed in pCS2+ with an N-terminal myc tag. The stable line was made by cloning *atoh7* promoter (Zolessi et al., 2006) into Tol2 zTRAP vector (42236; Addgene). MOs were obtained from Gene Tools. *Xenopus* Hermes and control MOs were conjugated to FITC and designed exactly as described previously by Zearfoss et al. (2004). *Xenopus* hermes a-MO: GCCCACCGAGGAGTCTGGCTTGTAC and *Xenopus* hermes b-MO: ATGAGCGGCATCAAGTCAGACACGG were injected at 2.5 ng each. The following zebrafish MOs and concentrations were used: hermes1a (rbpms2b), CTTGACACTCATCTTGTGCGTAAAC, 8 ng; hermes1b (rbpms2a), TTCAGACTCATTGTGTAACTTTAAC, 8 ng (both MOs have been verified previously; Hörnberg et al., 2013); and nrp1a, GAATCCTGGAGTTCGGAGTGCGGAA, 190 pg.

### Zebrafish embryo injection

MOs were injected into the yolk of zebrafish embryos at the one- to two-cell stage. Embryos were lined up on a plastic dish against a glass slide in a medium-free environment. Injections were performed using 0.78 mm needles pulled with a needle puller (1.0 mm OD × 0.78 mm, Harvard Apparatus; puller: Pul-1, World Precision Instruments) and 0.5–2 nl of volume was pressure injected using an air-pressure injector (Picospritzer II; Intracel).

### Translating ribosome affinity purification (TRAP)

Heads were dissected from 72 h postfertilization (hpf) atoh7:rpl10a-GFP and atoh7:gap-GFP embryos (*n* = 75/condition) in E3 embryo medium containing cycloheximide (100 μg/ml; Sigma-Aldrich) and MS222, snap-frozen on dry ice, and stored at −80°C. Tissues were homogenized in lysis buffer (20 mm Tris-HCl, pH 7.4, 5 mm MgCl_2_, 150 mm KCl, 1% NP40, 1 U/μl RNase OUT) and complete EDTA-free protease inhibitor cocktail supplemented with cycloheximide (100 μg/ml), incubated at 4°C for 30 min, centrifuged at 20,000 g for 20 min at 4°C. The supernatant was then collected and 10% of the extract was kept for total RNA extraction. The rest of the extract was then used for ribosome–mRNA complex immunoprecipitation with an anti-GFP antibody (catalog #ab6556, RRID:AB_305564; Abcam) preconjugated to Protein G magnetic beads (Dynabeads Protein G, 10004D; Life Technologies). After four washes with lysis buffer, the total RNA was extracted from the ribosome–mRNA complex using an RNeasy mini kit (Qiagen), followed by in-column DNase treatment to remove genomic DNA contamination. The RNA quality and quantity was then measured on the 2100 Bioanalyser System (Agilent Technologies) and the atoh7:gap-GFP condition allowed us to verify the immunoprecipitation specificity. cDNA synthesis was then performed using the Superscript III first-strand synthesis system (Invitrogen) and quantified with a Light Cycler 480 (Roche).

### *Xenopus* embryo injection

Embryos were injected as described previously (Vignali et al., 2000). Injections were performed at the four- or eight-cell stage in both dorsal animal blastomeres. Embryos were dejellied with 2% cysteine (Sigma-Aldrich) in 1 × MBS, pH 8, rinsed 3 × in 0.1 × MBS, and lined up on a grid in 4% Ficol (Sigma-Aldrich) in 0.1 × MBS, 1% penicillin (100 U/ml), streptomycin (100 μg/ml), and fungizone 0.25 μg/ml (PSF, Invitrogen). Then, 5 ng (2.5 each) of HeMOs was injected using glass capillary needles (1.0 mm outer diameter × 0.5 mm; Harvard Apparatus) and a microinjector (Picospritzer; General Valve).

### *Xenopus* Retinal explant cultures

Glass-bottom dishes (50 mm; Matek) were coated overnight at 20°C or at room temperature (RT) for a minimum of 3 h with poly-l-lysine (10 μg/ml) in double distilled H_2_O (ddH2O), followed by coating with laminin (10 μg/ml; Sigma-Aldrich) in L-15 medium (Invitrogen) for 1 h at RT. Embryo jelly coats were removed using forceps. All embryos were washed 3 × in 0.1 × MBS with 1% PSF to remove bacteria. Embryos were placed on a Sylgard-coated dish in a 1:1 mixture of 60% L-15 culture medium (60% L-15 in ddH2O and 1% PSF, pH 7.6–7.8) and MS222. Anesthetized embryos were pinned down with custom-made pins and the eye dissected out using dissection pins. Whole eye or eye pieces were then washed in 60% L-15 and plated on precoated dishes containing 60% L-15 culture medium with the lens side up. Dishes were incubated at 20°C for 12–24 h depending on the experiment.

### Collapse assays

Stage 28–40 retinal explants were cultured for 24 h at 20°C. For Sema3A collapse assay, cultures were treated with either 450 ng/ml recombinant human Sema3A Fc chimera (R&D Systems) in 0.1% protease-free bovine serum albumin (BSA; Sigma-Aldrich) or 0.1% BSA for 10 min, followed by fixation in 4% paraformaldehyde (PFA) containing 15% sucrose (Sigma-Aldrich) in 1 × PBS. All growth cone collapse was counted blind. Collapse was quantified as growth cones lacking lamellipodia and having two or fewer filopodia.

### Lipophilic dye labeling and imaging

Topographic analysis was done at 5 d postfertilization (dpf), when the tectum is first fully innervated. MO-injected zebrafish embryos were fixed with 4% PFA at 5 dpf and kept at 4°C for a minimum of 24 h. Embryos were pinned down on a custom-made Sylgard plate in 1 × PBS. The different quadrants in the retina were pressure injected using a microinjector (Picospritzer; General Valve) and 0.5 mm needles pulled as described previously. Ventral retina was injected with 3,3′-dioctadecyloxacarbocyanine perchlorate (DiO; Invitrogen) dissolved in dimethylformamide (Sigma-Aldrich), and dorsal retina with 1,1′-dioctadecyl-3,3,3′,3′-tetramethylindocarbocyanine perchlorate (DiI; Invitrogen) dissolved in 100% ethanol (Sigma-Aldrich). After 12–24 h incubation in the dark at RT, embryos were examined under epifluorescence and only embryos with perfect targeting in the eye were used for further analysis. For visualization of labeled axons, both eyes were removed using dissection pins and embryos were mounted either dorsally or laterally in a custom-made glass bottom dish in 1.2% low-melting-point agarose (24–28°C gelling point; Promega). Images were acquired using a PerkinElmer Spinning Disk UltraVIEW ERS; Olympus IX81 Inverted microscope, and 20 × (0.45 numerical aperture, NA) or 60 × (1.2 NA) water-immersion objective. Images were acquired using Volocity 3D Image Analysis Software (RRID:SCR_002668; PerkinElmer). All analysis was performed blinded to the MO knock-down.

### Puromycin assay

Zebrafish embryos (48 hpf) were incubated in in E3 embryo medium containing 200 μg/ml puromycin (Sigma-Aldrich). Fish heads were dissected at 72 hpf and homogenized in RIPA buffer (Sigma-Aldrich) supplemented with Halt protease and phosphatase inhibitor cocktail (Invitrogen). Proteins were resolved by 10% SDS-PAGE and transferred to nitrocellulose membrane (Bio-Rad). Antibodies used were mouse anti-puromycin (1:1000, RRID:AB_2566826, catalog #MABE343; Millipore) and mouse anti-α-tubulin (1:10,000, RRID:AB_477582, catalog #T6074; Sigma-Aldrich) antibodies, followed by horseradish peroxidase-conjugated secondary antibody (RRID:AB_955439, catalog #ab6789; Abcam). Bands were detected using an ECL-based detection (GE Healthcare).

### Immunohistochemistry

Eye explant cultures from *Xenopus* were fixed in 4% PFA and 15% sucrose in 1 × PBS for 30 min, washed 3 × 10 min in 1 × PBS, and permeabilized for 5 min in 0.1% Triton (Sigma-Aldrich) in 1 × PBS. The explants were gently washed 3 more times in 1 × PBS and blocked for 30 min in 5% heat-inactivated goat serum (HIGS). Primary antibodies were diluted in 5% HIGS and added to the explants for 1 h at RT. The explants were then washed 3 × 10 in PBS before incubation with the secondary antibody in 1 × PBS for 45 min at RT. The explants were washed a final 3 times in 1 × PBS before mounted with FluorSave reagent (Calbiochem) or imaged directly in 1 × PBS.

For immunostaining on cryostat sections, zebrafish embryos were fixed for 1–2 h in 4% PFA at RT, rinsed 3 × in 1 × PBS, and put in 30% sucrose in 1 × PBS for a minimum of 30 min. Embryos were embedded in Tissue-TEK OCT compound (SAKURA) and quick frozen on dry ice or at −80°C. Transverse retina or brain sections with a 10 μm thickness were cut using a cryostat (CM3050S; Leica). Slides were washed 3 × 10 min in PBS and permeabilized in 0.2% Tween (Sigma-Aldrich) in 1 × PBS before blocked for 1 h in blocking buffer (0.1% BSA, 10% HIGS, 0.1% Triton, 1 × PBS). Primary antibodies were incubated overnight at RT in a humidified chamber. Slides were then washed 3 × in 1 × PBS and incubated with secondary antibody for 1 h at RT in a humidified chamber in the dark. Slides were washed a final 3 × 10 min in PBS, incubated with 1:10000 DAPI for 45 min in a humidified chamber at RT, drained off, and mounted with FluorSave reagent. All slides were imaged using a PerkinElmer Spinning Disk UltraVIEW ERS; Olympus IX81 Inverted microscope, and 20 × (0.45 NA) objective. The following primary antibodies were used: anti-myc-tag (1:5000, RRID:AB_303599, catalog #ab32; Abcam), anti-Zn5 (1:500, RRID:AB_10013770, catalog #zn-5; Zebrafish International Resource Center), and anti-Nrp1 (1:50; gift from H. Fujisawa).

## Results

### Hermes depletion disrupts topography of dorsal axons in OT and tectum

**Figure 1.**
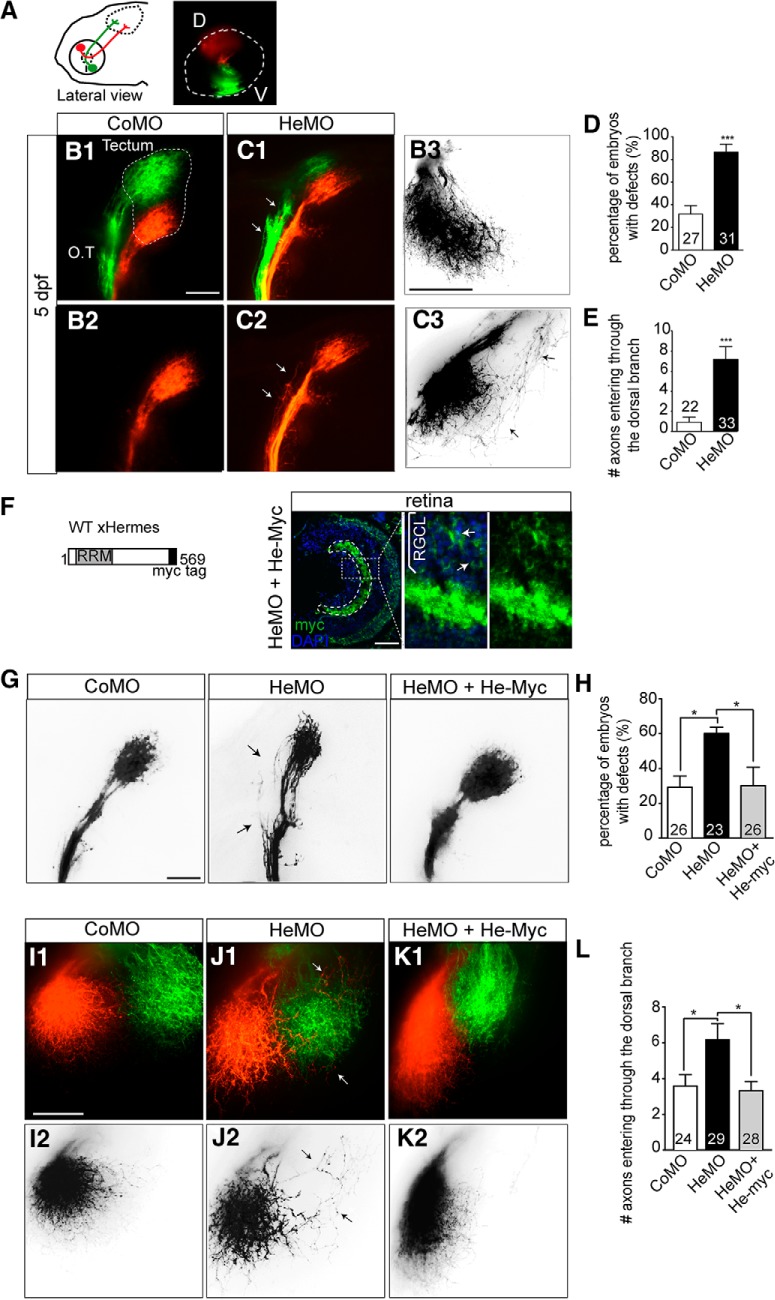
Knock-down of Hermes causes topographic guidance defects of RGC dorsal axons. Zebrafish embryos were fixed at 5 dpf and the eyes injected dorsally (D) with DiI (red) and ventrally (V) with DiO (green) to visualize the retinotectal projections (***A***). Whole-mount embryos injected with CoMO or HeMO were visualized in lateral (***B1***, ***B2***, ***C1***, ***C2***) or dorsal view (***B3***, ***C3***). Hermes-depleted embryos show misprojections of dorsal axons in the medial tract (***C1***, ***C2***, white arrows) that are not present in embryos injected with CoMO (***B1***, ***B2***). Quantifications show a significant increase of the percentage of embryos showing misprojections in the OT (***D***). HeMO-injected embryos show aberrant projection of dorsal axons entering the tectum through the ventral branch (***C3***) compared with CoMO (***B3***). Quantifications show that significantly more dorsal axons misroute and enter the tectum through the ventral branch in Hermes-depleted embryos compared with control (***E***). HeMO-injected embryos were coinjected with a construct expressing full-length myc-tagged *Xenopus* Hermes (He-myc; ***F***). After 72 hpf, immunostainings show a strong myc signal in the RGC layer (RGCL). Coinjection of He-myc rescues the dorsal axons misprojections in the OT observed in HeMO-injected embryos (***G***), with a significant reduction of the percentage of embryos with defects (***H***). In HeMO-injected embryos, some dorsal axons enter in the tectum through the medial tectum (***J1***, ***J2***) and this mistargeting is absent in He-myc-coinjected embryos (***K1***, ***K2***). Quantifications show a rescue of misprojecting dorsal axons in He-myc-injected embryos (***L***). Error bars indicate SEM. Numbers of embryos analyzed are indicated on bars. Scale bars, 50 μm.

Hermes knock-down can be achieved efficiently in zebrafish by injecting antisense MOs targeted to both *hermes1a* and *hermes1b* zebrafish transcripts (Hörnberg et al., 2013). We have shown previously that Hermes knock-down interferes with RGC synapse formation and arborization in the optic tectum (Hörnberg et al., 2013). To study the effect on topography, we used the lipophilic dyes DiI and DiO to label the dorsal and ventral part of the retina separately (Fig. 1*A*). Dye injections were performed at 5 dpf, when the tectum is first fully innervated. At this stage, dorsal and ventral axons are clearly segregated along the OT, with dorsal axons projecting through the lateral branch and ventral axons through the medial branch (Stuermer, 1988; Poulain and Chien, 2013). In control MO-injected embryos (CoMO), we saw the same result (Fig. 1*B1*,*B2*). In Hermes-depleted embryos (HeMO), however, dorsal axons were frequently observed ectopically in the medial branch of the OT (Fig. 1*C1*,*C2*), leading to a significant increase in the percentage of embryos with tract sorting defects compared with control embryos (*p* < 0.001, Fisher's exact test, Fig. 1*D*). Interestingly, ventral axons appeared to navigate correctly along the OT in HeMO-injected embryos, indicating that only dorsal axons were affected by Hermes depletion (Fig. 1*C1*,*C2*). In control embryos, dorsal axons reached the tectum through the lateral branch and projected directly to their target area in the medial tectum (Fig. 1*B3*). In HeMO-injected embryos, however, dorsal axons frequently entered the tectum aberrantly through the medial branch and then followed a circuitous route to the medial tectum (Fig. 1*C3*). In CoMO larvae, few if any axons made guidance errors on entering the tectum, whereas in HeMO embryos, there were many (control: 0.91 ± 0.50, HeMO: 7.20 ± 1.27, *p* < 0.0001, Mann–Whitney test; Fig. 1*E*).

In MO-based studies, it is critical to test the specificity of the induced phenotype. We therefore performed a rescue experiment by injecting a construct expressing a myc-tagged *Xenopus laevis* Hermes sequence (He-myc) into HeMO-injected zebrafish embryos (Fig. 1*F*). *Xenopus* Hermes mRNA has a high sequence similarity to zebrafish, but is not targeted by the zebrafish HeMOs. We first confirmed the construct expression by coronal sections of HeMO and He-myc-coinjected embryos showing strong Myc expression in the RGC layer (Fig. 1*F*). We next examined the retinotectal topography of Hermes morphants at 5 dpf with or without coinjection of He-myc. Coinjection of He-myc in HeMO-injected embryos significantly reduced the amount of embryos with misprojected dorsal axons compared with embryos injected with HeMO only, resulting in a percentage similar to control (*p* < 0.05, Fisher's exact test; Fig. 1*G*,*H*). Likewise, expression of He-myc was found to reduce the D–V sorting defect in the optic tectum observed after Hermes knock-down (*p* < 0.05, one-way ANOVA, Fig. 1*I–L*). Therefore, injection of *Xenopus* Hermes rescues the misrouting of dorsal axons observed in HeMO-injected zebrafish embryos, indicating that the phenotype observed in these embryos is specific to Hermes depletion.

### Hermes negatively regulates the translation of specific mRNAs in RGCs

**Figure 2.**
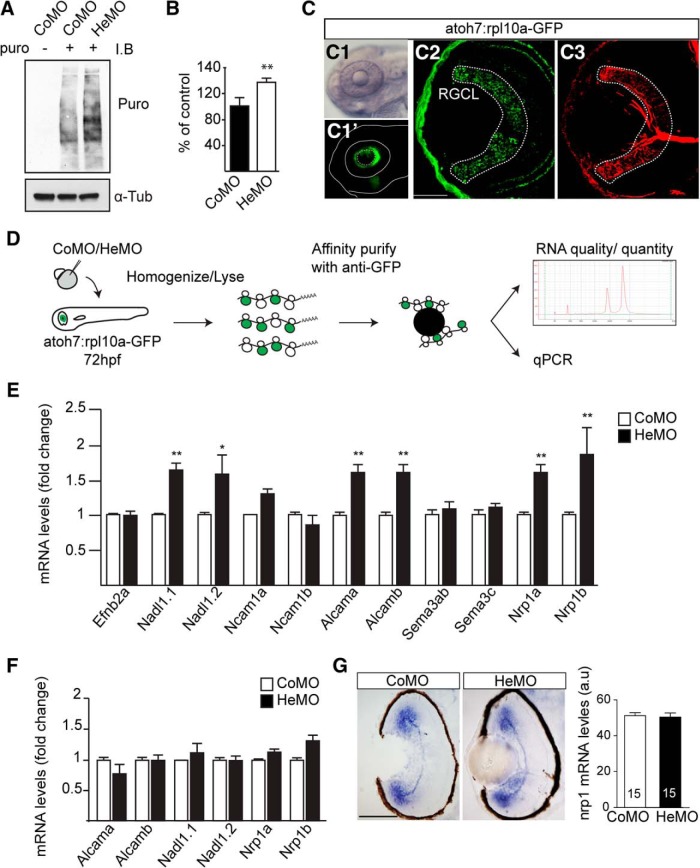
Hermes exerts a negative translational control of specific mRNAs in RGCs. Increase in puromycin incorporation detected by Western blotting in the Hermes-depleted condition compared with control (***A***, ***B***). GFP expression is restricted to the eye in the atoh7:rpl10a-GFP transgenic line (***C1***, ***C1′***), with a positive signal in Zn-5 positive RGCs and photoreceptors (***C2***, ***C3***). atoh7:rpl10a-GFP embryos at 72 hpf, injected with CoMO or HeMO, were homogenized and immunoprecipitations against GFP were performed on lysates. The total RNA was then extracted from the ribosome–mRNA complexes, analyzed by bioanalyzer, and quantified by quantitative RT-PCR (***D***). Quantifications show an increase of *nadl1.1*, *nadl1.2*, *alcama*, *alcamb*, *nrp1a*, and *nrp1b* mRNAs bound to ribosomes in absence of Hermes (***E***). Quantifications of total RNAs input show no difference between CoMO and HeMO conditions (***F***). *nrp1 in situ* hybridization on 72 hpf retinal transverse sections shows no difference between HeMO- and CoMO-injected embryos (***G***). Quantifications of signal intensity show no difference in *nrp1* expression in HeMO compared with CoMO (***G***). mRNA levels were calculated by using the formula 2^−ΔΔCt^ with β-actin mRNA as a calibrator (***E***, ***F***). Error bars indicate SEM. Numbers of embryos analyzed are indicated on bars. Scale bars: ***C2***, ***C3***, 30 μm; ***G***, 100 μm.

To investigate whether Hermes acts to repress or enhance translation in the zebrafish nervous system, we decided to quantify the global protein synthesis using puromycin incorporation into nascent peptide chains (Lin et al., 2009). Western blot analysis of 72 hpf embryo brain and eye extracts showed a significant increase of puromycin immunoreactivity in HeMO-injected embryos compared with control (Mann–Whitney test, Fig. 2*A*,*B*). This increase, indicative of a rise in protein synthesis, suggests that Hermes normally acts as a translational repressor.

We next sought to determine which mRNAs were specifically repressed in RGCs after Hermes depletion using a riboTRAP approach. We generated a zebrafish transgenic line in which the ribosomal protein rpl10a was tagged with GFP and its expression was under the control of the atoh7 promoter (atoh7:rpl10a-GFP; Tryon et al., 2013; Fig. 2*C*). As expected, rpl10a-GFP expression was limited to the eye (Fig. 2*C1*,*C2*), with positive signal detected in the RGC (visualized with Zn-5 staining; Fig. 2*C2*,*C3*) and photoreceptor layers (Fig. 2*C1–C3*). To isolate ribosome-associated mRNAs from RGC cell bodies and axons, rpl10a-GFP immunoprecipitation was performed on eyes and brains from HeMO- or CoMO-injected embryos collected at 72 hpf (Fig. 2*D*). We then performed a quantitative RT-PCR analysis on 11 genes known to be involved in sensory topography map formation (Fig. 2*E*). Six housekeeping genes were selected for normalization. Of the 11 genes analyzed, six showed a significantly higher amount of ribosome-bound mRNA after Hermes knock-down compared with control, with no change observed for the others (*n* = 3 experiments, Mann–Whitney test; Fig. 2*E*). The six genes that showed increased translation in the HeMO condition were found to correspond to the zebrafish duplicated genes of the mammalian orthologs of the cell adhesion molecules L1cam (*nadl1.1*, *nadl1.2*) and ALCAM (*alcama*, *alcamb*) and the guidance receptor Neuropilin1 (*nrp1a*, *nrp1b*) (Fig. 2*E*).

It is possible that the increased ribosome association of these six mRNAs is due to an overall increase on their abundance. To investigate this, we first did a quantitative RT-PCR analysis for the six mRNAs from total RNA extracts (Fig. 2*F*). These results showed no significant difference in the amounts of message between CoMO and HeMO conditions for the 6 mRNAs analyzed (*n* = 3 experiments, Mann–Whitney test), indicative of no difference in overall mRNA abundance. We also performed *in situ* hybridizations for *nrp1* mRNA on transverse sections of 72 hpf HeMO- and CoMO-injected embryos (Fig. 2*G*). Again, we observed no obvious change in the localization and quantification of signal intensity (n.s, Mann–Whitney test; Fig. 2*G*). Together, these results showed a specific function for Hermes in RGCs as a negative translation regulator of restricted target mRNAs.

### Hermes depletion increases Nrp1 in RGC growth cones and confers premature sensitivity to Sema3A

**Figure 3.**
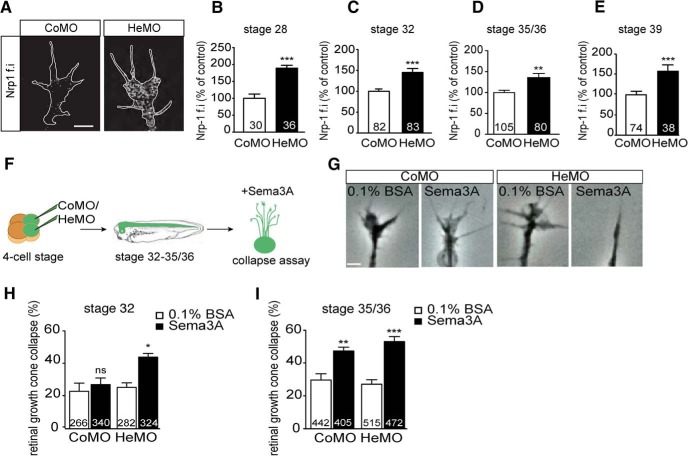
Hermes depletion increases Nrp1 protein in axons and induces an earlier response to Sema3A. Immunostainings on stage 32 *Xenopus* retinal explants show increased Nrp1 expression on Hermes-depleted growth cone compared with control (***A***). Quantifications show a significant increase in Nrp1 signal intensity in the growth cone at stage 28 (***B***), stage 32 (***C***), stage 35/36 (***D***), and stage 39 (***E***) embryos. Eye explants were cultured from stage 32 and 35/36 *Xenopus* embryos for 24 h. Sema3A was added for 10 min before fixation and the percentage of collapsed growth cones was determined (***F***). BSA was used as a control. Examples of collapse response of control and Hermes-depleted growth cones (***G***). At stage 32, Hermes-depleted growth cones display a significantly higher collapse response to Sema3A compared with control (***H***). This increase in collapse response is not present at stage 35/36 (***I***). Error bars indicate SEM. Numbers of growth cones analyzed are indicated on bars. Scale bars, 100 μm.

Previous *Xenopus laevis* studies have shown precise, intrinsic regulation of Nrp1 expression in RGCs during development, with a sharp stage-dependent increase of the protein in axons and growth cones (Campbell et al., 2001). Delaying the expression of Nrp1 by MO injection was found to induce pathfinding errors in the OT by interfering with the growth cone's responsiveness to Sema3A (Baudet et al., 2011). We hypothesized that Hermes functions to restrict Nrp1 levels in RGC growth cones, thereby regulating their sensitivity. Using *Xenopus* retinal explants, we examined Nrp1 expression levels in RGC axons and growth cones (Fig. 3*A–E*). Quantification of Nrp1 immunoreactivity showed a significant increase in Hermes-depleted RGC growth cones compared with control growth cones (Fig. 3*A*). This increase was found throughout RGC axon development (Fig. 3*B–E*).

To test whether higher levels of Nrp1 in growth cones would be sufficient to induce a precocious cue response, we tested the sensitivity to Sema3A by performing a collapse assay (Fig. 3*F–I*). As observed previously, retinal explants from stage 32 cultured for 24 h were not sensitive to Sema3A (Fig. 3*H*; Campbell et al., 2001; Baudet et al., 2011), whereas a significant Sema3A-induced collapse was seen in explants from stage 35/36 embryos (Fig. 3*I*). In contrast, explants from stage 32 HeMO morphant eyes displayed a significant Sema3A-induced collapse response (Fig. 3*H*), which was also present at stage 35/36 (Fig. 3*I*). These results show that the regulation of intrinsic Nrp1 levels is Hermes dependent in RGCs and controls growth cone cue sensitivity.

### Nrp1 rescues the guidance errors of dorsal axons in Hermes-depleted RGCs

**Figure 4.**
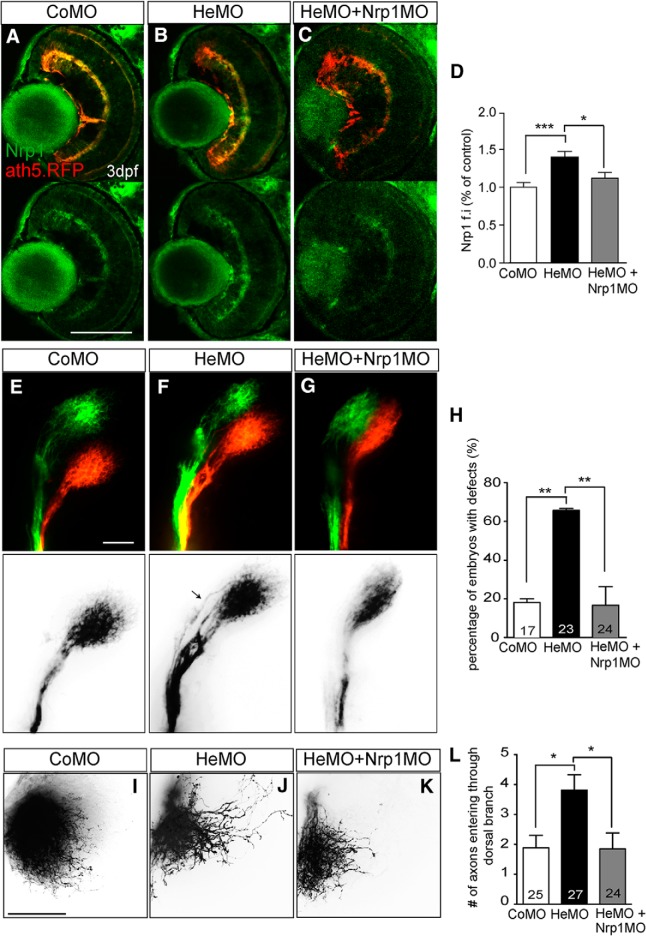
Restoring Nrp1 levels rescues the dorsal axon topography defect in Hermes-depleted embryos. Shown is Nrp1 immunostaining on retina transverse sections from atoh7:gapRFP zebrafish embryos injected with CoMO (***A***), HeMO (***B***), or HeMO+Nrp1MO (***C***). Quantifications show a significant increase of Nrp1 signal intensity in the HeMO condition compared with CoMO and HeMO + Nrp1MO (***D***). Lateral view of whole-mount DiI- and DiO-injected retina from CoMO- (***E***), HeMO- (***F***), and HeMO + Nrp1MO (***G***)-injected embryos. HeMO + Nrp1MO-coinjection showed a significant reduction of the percentage of embryos with mistargeting in the OT compared with HeMO (***H***). DiI-labelling of dorsal axons in the tectum of CoMO (***I***), HeMO (***J***), and HeMO + nrp1MO (***K***) injected embryos. Quantifications show a rescue of misprojecting dorsal axons in HeMO+nrp1MO injected embryos (***L***). Error bars indicate SEM. Numbers of embryos analyzed are indicated on bars. Scale bars: ***A***–***C***, 100 μm; ***E***–***G***, ***I***–***K***, 50 μm).

The above findings suggest that the missorting of dorsal axons in the OT of Hermes-depleted embryos may be caused by dysregulated translation of Nrp1, leading to premature and aberrant expression of Nrp1 in retinal growth cones. To examine this idea, we first investigated whether Nrp1 expression was increased *in vivo* in Hermes-depleted eyes. As expected, immunostaining on 72 hpf zebrafish retina showed a significant increase in Nrp1 expression in Hermes-depleted embryos compared with control (Kruskal–Wallis test, Dunn's multiple-comparisons test; Fig. 4*A–D*).

We next tested whether the guidance errors observed in Hermes morphants were caused by the elevated levels of Nrp1 by reducing the expression of Nrp1 in HeMO embryos. To do this, we used a low dose of Nrp1MO (190 pg; Fig. 4*C*,*D*). As shown previously, Hermes depletion alone caused a significant increase in the percentage of embryos with missorted dorsal axons compared with control embryos at 5 dpf (*p* < 0.01, χ^2^ test; Fig. 4*E–H*). However, coinjection of 190 pg of Nrp1MO reduced the percentage of embryos with guidance errors in the OT to 28%, significantly lower than HeMO-injected embryos and similar to the percentage of missorting found in control-injected embryos (*p* < 0.01, χ^2^ test; Fig. 4*G*,*H*). The rescue of tract missorting is reflected in the targeting of dorsal axons in the tectum, which showed a significant decrease in Nrp1MO-coinjected embryos compared with embryos injected with HeMO alone (*p* < 0.05, Kruskal–Wallis test, Dunn's multiple-comparisons test; Fig. 4*I–L*). These results demonstrate that the topographic errors of dorsal axons in Hermes-depleted embryos are at least partially due to dysregulated Nrp1 expression.

## Discussion

In this study, we show that the RBP Hermes regulates the sorting of RGC axons in the OT. Our data revealed that Hermes functions as a translational repressor by negatively regulating the translation of specific mRNAs in RGCs. We show that Hermes is important for the intrinsic control of Nrp1 expression in the growth cone and thus its sensitivity to extracellular cues such as Sema3A. Furthermore, we found that reducing the level of Nrp1 in Hermes-depleted embryos was sufficient to rescue the guidance errors of dorsal axons. Therefore, this study demonstrates a novel role for RBP-mediated posttranscriptional control in topographic map formation by regulating the precise stage-dependent changes in growth cone responsiveness. Our results indicate that Hermes is a repressor of *nrp1* mRNA translation, either directly or indirectly, which controls Nrp1 expression temporally in RGC growth cones. Interestingly, the mammalian ortholog of Hermes, RBPMS2, has also been shown to bind to *nrp1* mRNA (Farazi et al., 2014).

How does misregulation of Nrp1 expression lead to topographic presorting defects? As is the case for the bulb-olfactory system, it is possible that Nrp1 expression enables RGC dorsal axons to respond to a repulsive cue topographically expressed by either an intermediate target along the path or by the ventral RGC axons (Imai et al., 2009). Several Semaphorins are expressed in the RGC layer or along the OT (Kuwajima et al., 2012). Surprisingly, knock-down of class III Semaphorins does not seem to affect the topographic projections of RGC axons *in vivo* (data not shown). However, the vascular endothelial growth factor 164 (VEGF-164), another Nrp1 ligand, displays an expression along the RGC axonal pathway and mice lacking VEGF-164 show defasciculation of the OT, indicating a potential role in tract sorting (Erskine et al., 2011). A second possibility is that Nrp1 regulates homotypic fasciculation between RGC axons. Indeed, Nrp1 has been shown to regulate axon–axon interactions in motor neurons (Huettl et al., 2011). Therefore, Nrp1 expression in RGC growth cones may play a role during homotypic and/or heterotypic axon–axon cell-contact recognition. A third possibility relates to the fact that the path of dorsal RGC axons is refined during development (Poulain and Chien, 2013). This would result from degeneration of mistargeted RGC axons in zebrafish between 50 and 72 hpf (Poulain and Chien, 2013). Consistent with this suggestion is the finding that Nrp1 is required for axonal pruning during development (Bagri et al., 2003). Further studies are needed to distinguish between these hypotheses and to define the molecular mechanisms through which Nrp1 expression affects axon sorting in the OT.

We have shown previously that Hermes depletion reduces RGC arbor complexity in zebrafish using single axon tracing and analysis (Hörnberg et al., 2013). The topographic origin of the axons was not known in that previous study, so the axon-sorting errors described here were not detected. It is possible that missorted dorsal axons have a more severe arborization phenotype. The elevated Nrp1 levels at 3 dpf coincides temporally with the first observed alterations in branch dynamics in Hermes morphants (Hörnberg et al., 2013), suggesting that the increased Nrp1 levels could play a role in the reduced arborization. Indeed, Sema3A was found to increase RGC branch formation *in vitro* (Campbell et al., 2001) and Nrp1-Sema3A signaling regulates layer-specific branching in the mouse cerebellum (Cioni et al., 2013). However, in zebrafish, branching contributes to pathfinding via selective stabilization of branches formed toward the topographically correct target zone (Simpson et al., 2013). In Hermes-depleted embryos, branch stability is unaffected, suggesting that Hermes may regulate topographic sorting and arborization via different mechanisms.

We have shown that Hermes expression in RGCs is critical to restraining the levels of Nrp1 expression in the growth cone through the different developmental stages and that the experimental knock-down of Hermes leads to the precocious upregulation of Nrp1, increased sensitivity to Sema3A, and tract-sorting errors. There are several questions that our study has not been able to resolve. One of these is whether the tract-sorting defect observed in the absence of Hermes results from precocious Nrp1 expression during the early stages of tract formation or the continuously elevated levels of Nrp1 throughout axon elongation in the OT. To resolve this question, it will be essential to gain precise temporal control of Nrp1 expression. Another question concerns other factors that control Nrp1 expression. For example, it is known that Nrp1 levels rise after stage 32 (32 hpf) even though Hermes continues to be expressed in RGCs (Hörnberg et al., 2013). The onset of Nrp1 expression in RGCs is indirectly regulated by the microRNA miR-124 targeting of RE1 silencing transcription factor corepressor 1 (CoREST), a transcriptional corepressor (Baudet et al., 2011). The expression of miR-124 is upregulated and CoREST downregulated over time, allowing the increase of Nrp1 expression. The function of the mammalian ortholog of Hermes, RBPMS2, in RGCs has not been characterized; however, transcriptome analyses after optic nerve injury reveal a downregulation of RBPMS2 concomitant with an increase of Nrp1 levels in RGC axons (Yasuda et al., 2014; Han et al., 2015; Yasuda et al., 2016) and rescue of RGC degeneration can be achieved by decreasing Nrp1 function (Shirvan et al., 2002). The parallels between these findings and those described herein suggest that this protective effect on mammalian optic axons may be achieved by similar molecular mechanisms. Finally, our study does not address whether the translation regulation of Hermes occurs in the axon and/or the cell body. Previous work has not detected the presence of *nrp1* mRNA in RGC axons nor its translation (Zivraj et al., 2010; Shigeoka et al., 2016); however, it may have escaped detection due to low mRNA abundance and tight translational regulation. Hermes protein is expressed in RGC axons and is detected in growth cones (Hörnberg et al., 2013), opening the possibility of a local function. It is worth noting that the two other Hermes mRNA targets identified in this study, Nadl1 and Alcam, have been identified in growth cones (Zivraj et al., 2010; Thelen et al., 2012). Both have been implicated in the mapping of ventral axons in the mouse visual system (Demyanenko and Maness, 2003; Buhusi et al., 2008; Buhusi et al., 2009), raising the possibility that Hermes may regulate additional aspects of pathfinding locally via its other mRNA targets.

In summary, we have shown that Hermes acts as a translational repressor of Nrp1 expression in RGCs and that this regulation is important for correct axonal sorting in the OT. Although important questions remain, the evidence that we provide demonstrates a new RNA-based molecular mechanism that operates during axonal navigation to regulate specific guidance receptors and affect accurate pathfinding decisions.
